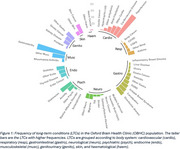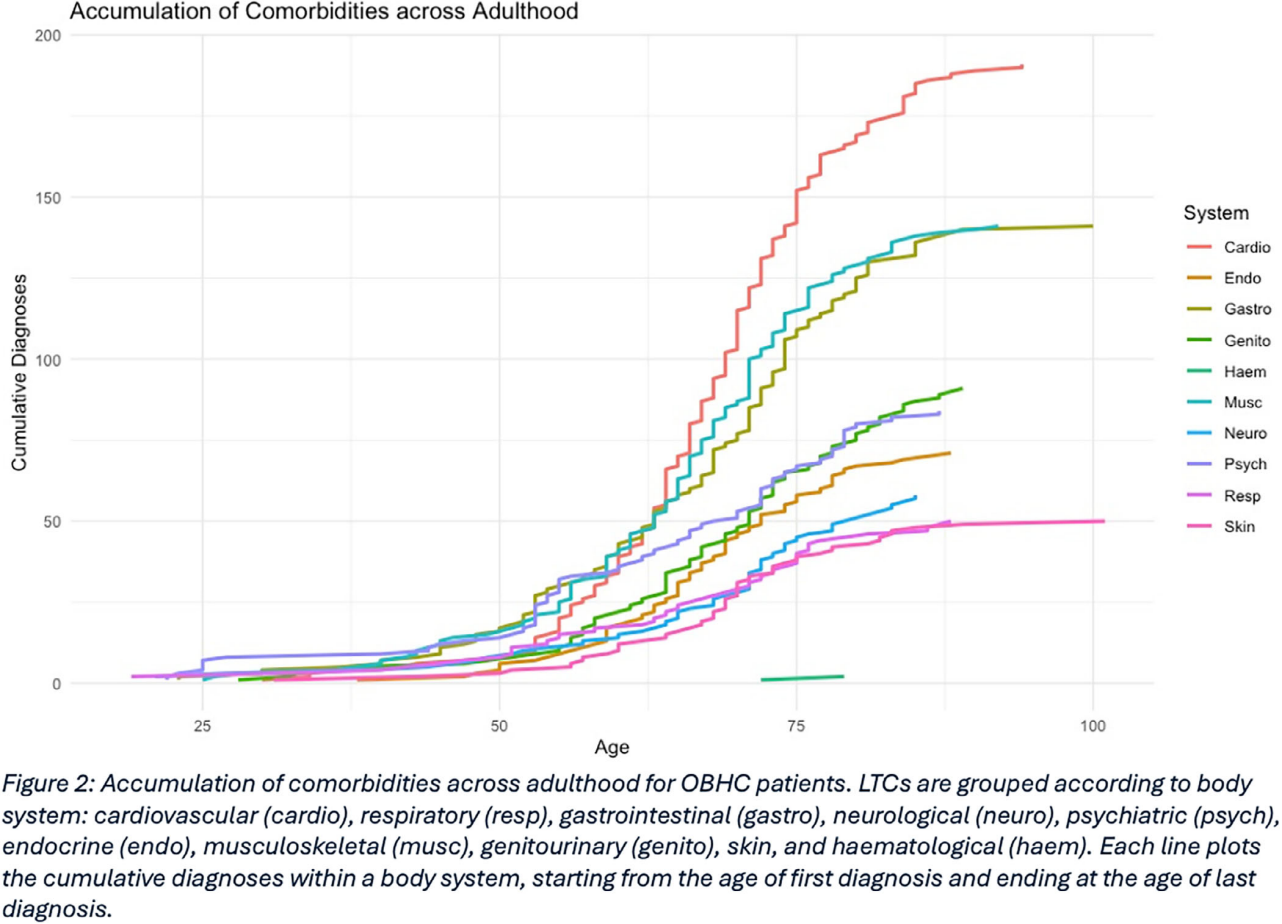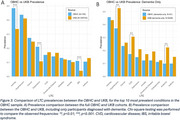# Characterisation of long‐term conditions in a memory clinic population: A UK Biobank comparison study

**DOI:** 10.1002/alz70860_105761

**Published:** 2025-12-23

**Authors:** Grace Gillis, Jasmine Blane, Raihaan Patel, Sarah Fynes‐Clinton, Inga Farafontova, Dansheela Makan, Lola Martos, Vanessa Raymont, Sana Suri, Ludovica Griffanti, Clare E Mackay

**Affiliations:** ^1^ University of Oxford, Oxford, Oxfordshire, United Kingdom; ^2^ Oxford Health NHS Foundation Trust, Oxford, Oxfordshire, United Kingdom; ^3^ University of Oxford, Oxford, England, United Kingdom

## Abstract

**Background:**

Multimorbidity across the lifespan, especially during critical age windows, is associated with increased dementia risk. In this study, we sought to characterise the accumulation of long‐term conditions (LTCs) in a real‐world memory clinic population at the Oxford Brain Health Clinic (OBHC). We contextualise these prevalences with comparison to UK Biobank (UKB).

**Method:**

By 2025, medical histories extracted from primary care records were available on the OBHC Research Database for 190 NHS memory clinic patients. 50 LTCs or categories of LTCs were prioritised during extraction and grouped according to body system (Figure 1), recording the first time each diagnosis was made. To align with previous comorbidity lists (Patel *et al.*, medRxiv, 2024), some conditions were merged (e.g., cancers, types of arthritis), and others were extracted from free‐text (e.g., anaemia, macular degeneration, osteoporosis, prostate, sleep disorders, hyperlipidaemia), resulting in a list of 31 LTCs for comparison. In line with the OBHC cohort, UKB participants younger than or deceased before age 65 were excluded. A supplementary comparison was performed including only those with a dementia diagnosis, excluding those from UKB who were diagnosed before age 65; only pre‐dementia LTCs were considered.

**Result:**

On average, OBHC patients had 4 comorbid diagnoses before attending their memory clinic appointment; osteoarthritis and hypertension were most common, with a relative prevalence of 46.8% and 43.7%, respectively (Figure 1). In early adulthood, psychiatric conditions were most prevalent in OBHC patients, but cardiovascular conditions accumulated most rapidly across midlife to become the most prevalent (Figure 2). Of the 10 most prevalent LTCs in the OBHC, arthritis, depression, and IBS were more prevalent than in UKB (Figure 3A). OBHC dementia patients had significantly lower prevalences of hypertension, cardiovascular disease, and anaemia than UKB dementia patients (Figure 3B).

**Conclusion:**

Although the rankings of conditions between cohorts were largely similar, we found potentially important differences in the prevalence of LTCs between a large population cohort and a real‐world patient sample. Ascertaining comparable data across research and clinical cohorts is a harmonisation challenge that needs to be met so the impact of multimorbidity on brain health can inform the development of personalised interventions.